# Current status and prospects of endovascular treatment for intracranial vertebral artery aneurysms: A narrative review

**DOI:** 10.1097/MD.0000000000042265

**Published:** 2025-04-25

**Authors:** Yanming Qu, Jinlu Yu

**Affiliations:** a Department of Neurosurgery, Jilin City Hospital of Chemical Industry, Jilin City, China; b Department of Neurosurgery, the First Hospital of Jilin University, Changchun, China.

**Keywords:** aneurysm, endovascular treatment, intracranial vertebral artery, prognosis, review

## Abstract

Intracranial vertebral artery (VA) aneurysms are complex entities. Endovascular treatment (EVT) can be used to treat intracranial VA aneurysms. Nevertheless, managing these lesions with EVT remains challenging. Moreover, the suitability of EVT for every type of intracranial VA aneurysm has not been fully confirmed. Therefore, we conducted a needed review of the current literature and our experience summarizing the current status of and advancements in EVT in the management of intracranial VA aneurysms. In our review, several issues are discussed, including the anatomy and anomalies of the intracranial VA, the classification and natural history of intracranial VA aneurysms, indications and techniques for EVT in the management of intracranial VA aneurysms, and the outcomes of and complications experienced by patients who undergo EVT. A flowchart describing EVT options for dissecting intracranial VA aneurysms derived from the findings of this review and our experience is provided. The key to successful EVT is preservation of the posterior inferior cerebellar artery and avoidance of injury to any brainstem perforators. Currently, intracranial VA reconstruction via flow diverter deployment plays an important role in achieving successful treatment. For appropriate cases, both reconstructive and deconstructive EVT can result in good patient outcomes. However, EVT-related complications should be considered. If management of complex intracranial VA aneurysms with EVT would be expected to disproportionally harm the patient, extracranial-intracranial bypass and aneurysmectomy are often necessary. In addition, new products and techniques that show promise for achieving successful EVT in the management of intracranial VA aneurysms are described.

## 1. Introduction

Intracranial vertebral artery (VA) aneurysms account for 0.5% to 3% of intracranial aneurysms and 15% of posterior circulation aneurysms.^[1]^ If an intracranial VA aneurysm ruptures or becomes symptomatic, treatment is necessary. Owing to their variable morphologies, intracranial VA aneurysms are complex. Moreover, if left untreated, they are associated with high disability and mortality rates. The presence of important neural tissue structures surrounding them and the perforating vessels that supply these structures make open surgical treatment of this condition difficult.^[2,3]^

Currently, endovascular treatment (EVT) is considered an attractive alternative for managing intracranial VA aneurysms.^[4]^ In particular, flow diverters (FDs) with a > 30% metal coverage rate have revolutionized the use of EVT for managing intracranial VA aneurysms by redirecting the blood flow of the parent artery and promoting thrombosis in the aneurysm.^[5]^ Owing to the heterogeneity of intracranial VA aneurysms, however, the optimal EVT technique for managing this condition has not been fully elucidated.^[6]^ Therefore, a review summarizing the current status and prospects of EVT for intracranial VA aneurysms on the basis of a literature review and our experience is necessary.

## 2. Methods and results

### 2.1. Literature search and strategy

PubMed was searched for English articles published from 1990 to April 1, 2025. The keywords included “intracranial VA anatomy and anomaly,” “intracranial VA aneurysm,” “intracranial VA dissection,” “intracranial vertebral dolichoectasia,” “endovascular treatment” and “open surgery” and were used alone or in relevant combinations. The types of articles searched included case reports, case series, cohort studies, randomized controlled trials and prior systematic reviews and meta-analyses. The reference lists of the identified articles were also manually searched for additional relevant articles.

### 2.2. Inclusion and exclusion criteria

**Figure 1.**
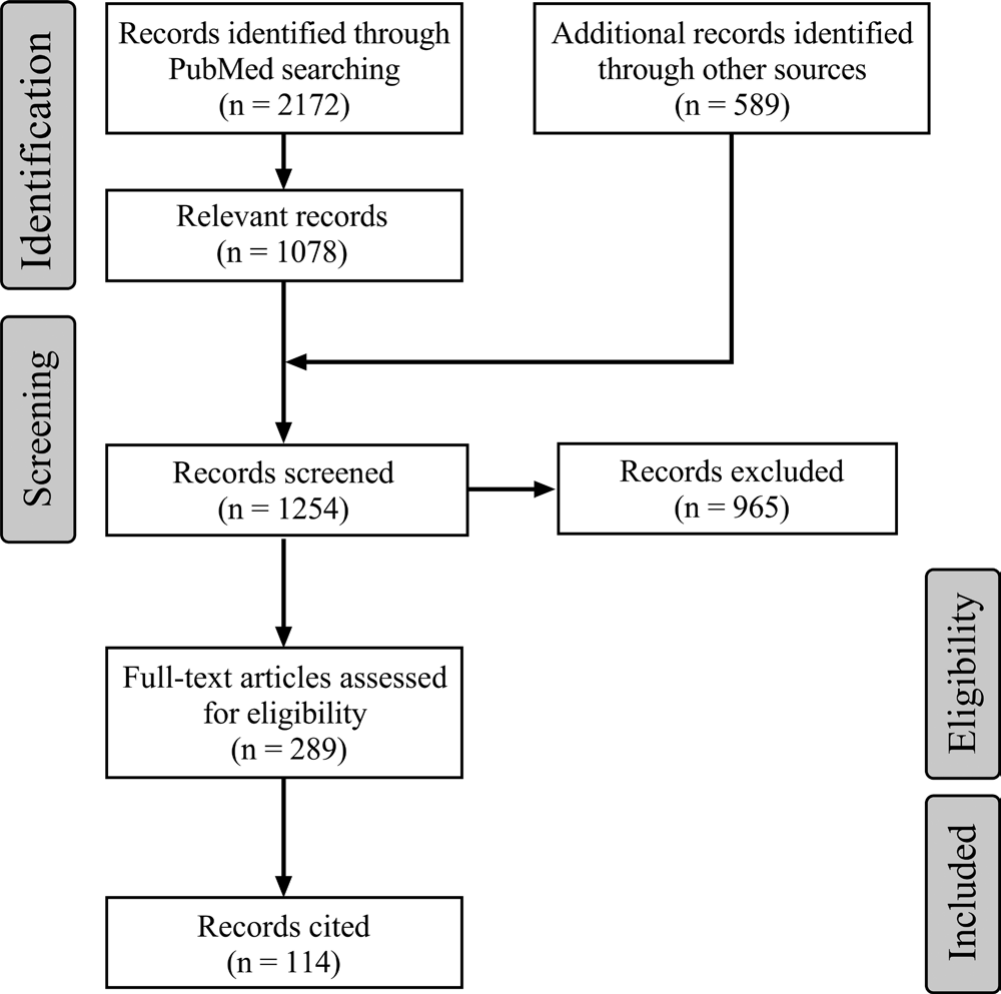
Flow chart of the literature search.

Articles were included if: the full text or sufficient information could be obtained; and they investigated “the anatomy and anomalies of the intracranial VA, the classification and natural history of intracranial VA aneurysms, and EVT and surgical treatment for intracranial VA aneurysms.” Articles without sufficient information were excluded. Articles published before 1990 were excluded, as modern EVT techniques became highly widespread after this date. After screening the articles, a total of 114 relevant manuscripts were retained. A flow chart of the literature collection process is shown in Figure 1.

## 3. Anatomy and anomalies of the intracranial VA

### 3.1. Angiographic anatomy

**Figure 2.**
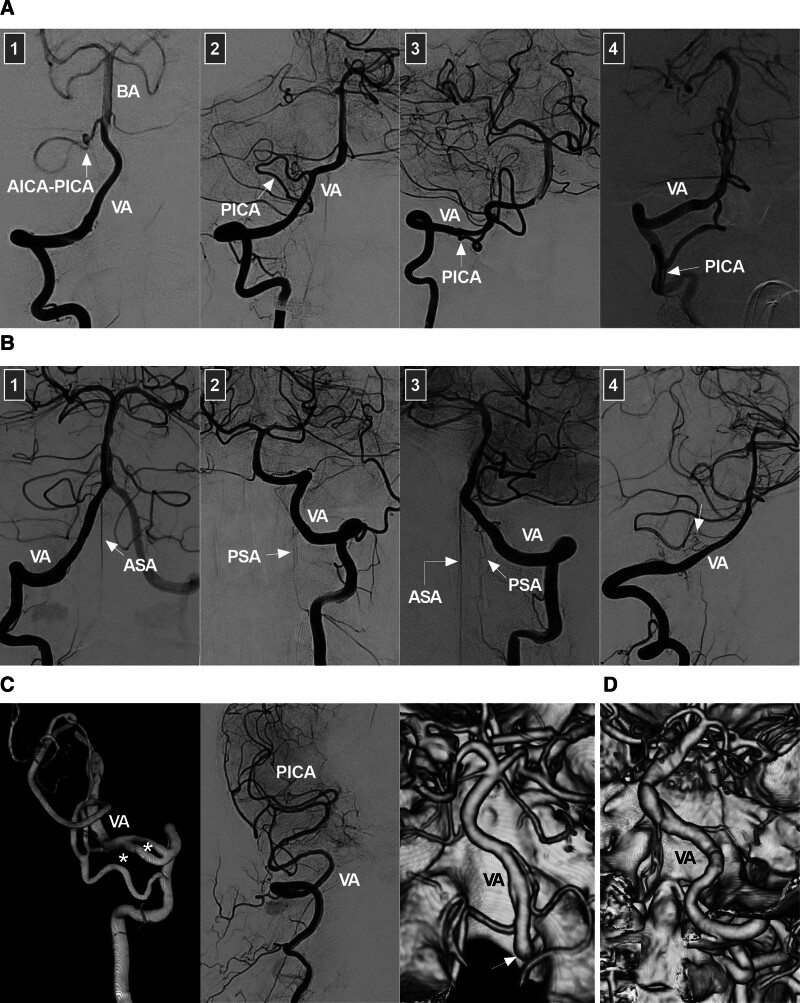
Intracranial VA anatomy and anomalies. (A) PICA anatomy: panel 1: DSA image showing the PICA and AICA sharing a common trunk (arrow); panel 2: DSA image showing the PICA (arrow) emerging from the distal VA; panel 3: DSA image showing the PICA (arrow) emerging from the intracranial proximal VA; panel 4: DSA image showing the PICA (arrow) emerging from the extracranial VA. (B) ASA and PSA anatomy: panel 1: DSA image showing the ASA (arrow) emerging from the VA; panel 2: DSA image showing the PSA (arrow) emerging from the VA; panel 3: DSA image showing the ASA and PSA (arrows) emerging from the VA; panel 4: DSA image showing a perforating artery (arrow) emerging from the VA. (C) VA anomaly: left panel: DSA image showing 2 fenestrations (asterisks) of the VA; middle panel: DSA image showing a VA terminating at the PICA; right panel: CTA image showing a VA emerging from the primitive hypoglossal artery (arrow). (D) CTA showing VA dolichoectasia, a form of vertebrobasilar dolichoectasia. AICA = anterior inferior cerebellar artery, ASA = anterior spinal artery, BA = basilar artery, CTA = computed tomography angiography, DSA = digital subtraction angiography, PICA = posterior inferior cerebellar artery, PSA = posterior spinal artery, VA = vertebral artery.

The intracranial VA begins at its dural penetration and terminates at the vertebrobasilar junction (VBJ), where it helps form the basilar artery (BA).^[7]^ This segment is also called the intradural VA, V4 segment or distal VA.^[8,9]^ The intracranial VA gives off several important branches, including the posterior inferior cerebellar artery (PICA), the largest branch (Fig. 2A), as well as the anterior spinal artery (ASA), the posterior spinal artery (PSA) and various perforating arteries (Fig. 2B). The ASA is formed by 2 small branches that arise from each VA,^[10]^ whereas the PSA may arise from the VA or PICA.^[11]^ The perforating arteries, which may originate from the VA, PICA or VBJ, are typically located in the region between the PICA and BA.^[11,12]^

### 3.2. Development and anomalies

The average length of the intracranial VA is 3 cm, and its average diameter is 3 mm.^[13,14]^ The left VA is often larger than the right VA.^[8,14]^ Intracranial VA anomalies included hypoplasia, fenestration, VA termination at the PICA (known as VA atresia), and VAs emerging from the primitive hypoglossal artery (Fig. 2C).^[14,15]^ Hypoplasia is defined as a VA diameter < 2 mm.^[16]^ Fenestrations at the distal V4 segment are often small; however, fenestrations from the distal V3 to proximal V4 segments are often large and can be described as arterial rings or VA duplications.^[15]^ VA dolichoectasia rarely occurs as a form of vertebrobasilar dolichoectasia (Fig. 2D).^[17]^ These anomalies of the VA may make EVT difficult.

## 4. Classifications of intracranial VA aneurysms

### 4.1. Morphology and pathology

**Figure 3.**
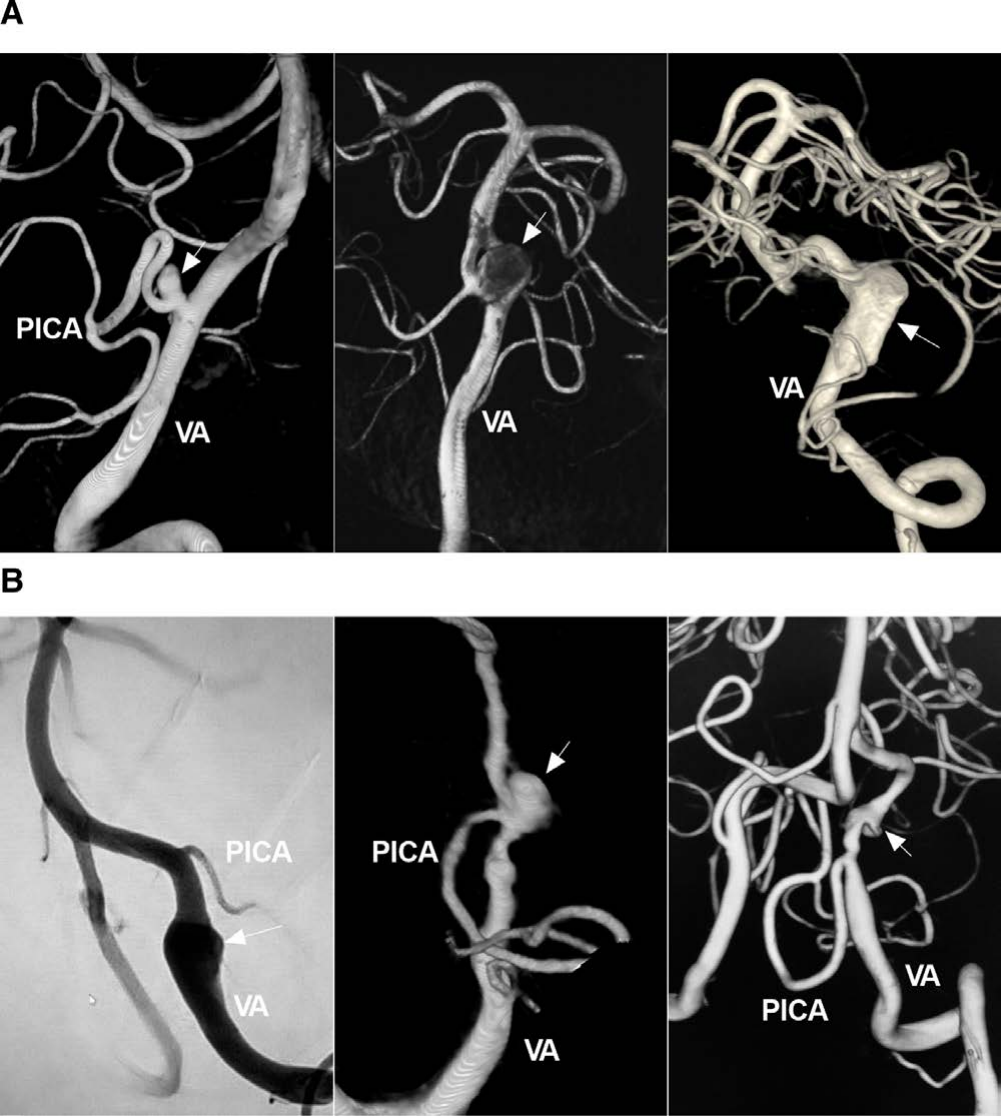
Types of intracranial VA aneurysms. (A) DSA images showing a saccular aneurysm (arrow) at the PICA-VA junction (left panel), a saccular aneurysm (arrow) in a fenestration at the VA terminus (middle panel), and a dissecting aneurysm (arrow) of the intracranial VA trunk (right panel). (B) DSA images showing a pre-PICA (below the PICA) VA trunk aneurysm (arrow) (left panel), a VA trunk aneurysm (arrow) with PICA involvement (middle panel), and a post-PICA (above the PICA) VA trunk aneurysm (arrow) (right panel). DSA = digital subtraction angiography, PICA = posterior inferior cerebellar artery, VA = vertebral artery.

On the basis of their shape, intracranial VA aneurysms can be divided into saccular and nonsaccular lesions. Saccular lesions are often bifurcation aneurysms located at the VA–PICA junction or VA fenestrations (Fig. 3A). Nonsaccular lesions are often dissected aneurysms, are located on the VA trunk or the fenestrated limb, and present with fusiform or lateralized dilatation that may coexist with stenosis (Fig. 3B).^[4,18,19]^

### 4.2. Location

Intracranial VA trunk aneurysms can be divided into 3 types according to the position between the aneurysm and the PICA origin: VA trunk aneurysms with PICA involvement, pre-PICA (below the PICA) VA trunk aneurysms, and post-PICA (above the PICA) VA trunk aneurysms (Fig. 3B).^[20]^ Post-PICA-VA trunk aneurysms are the most common, accounting for 60% of intracranial VA trunk aneurysms.^[1,20]^

### 4.3. Other classifications

According to investigators from the International Study of Unruptured Intracranial Aneurysms, intracranial VA aneurysms can be divided on the basis of size into small (<7 mm), medium (7–12 mm), large (>12–25 mm), or giant (> 25 mm) aneurysms.^[21]^ Additionally, intracranial VA aneurysms can be divided into asymptomatic, ruptured hemorrhagic, or unruptured aneurysms with mass effects or ischemia. Furthermore, intracranial VA aneurysms may be bilateral, mirror-like, and symmetric.^[22]^

## 5. Natural history of intracranial VA aneurysms

### 5.1. Saccular aneurysms

Ruptured saccular aneurysms at the VA–PICA junction have a potentially unfavorable course, and the risk of rebleeding is high if no treatment is implemented.^[23,24]^ The exact natural history of unruptured lesions is unclear. Small lesions may be stable, but large, irregular, symptomatic and growing lesions tend to rupture.^[25,26]^ According to the Unruptured Cerebral Aneurysm Study in Japan, for aneurysms at the VA–PICA junction, the rate of rupture per aneurysm per year is 0% among aneurysms < 10 mm in size, increasing to 3.5% for aneurysms ranging from 10 to 24 mm in size.^[27]^

### 5.2. Dissecting aneurysms

Ruptured dissecting intracranial VA aneurysms have a high rate of rebleeding, mostly within the first 24 hours, especially for those associated with “stenosis and dilation” and “lateral protrusion” and those at or proximal to the origin of the PICA.^[28,29]^ Asymptomatic lesions may have a benign natural history^[30,31]^; however, lesions with mass effects or associated with stroke have a poor natural history.^[32,33]^ A poor intracranial VA aneurysm natural history is associated with an aneurysm size > 10 mm.^[34]^ Fusiform intracranial VA aneurysms may originate from dissections, and asymptomatic, nonlarge chronic lesions have been shown to be stable within the first few years.^[5]^ Intracranial VA dolichoectasia may be characterized by dissection, and its natural history can vary. Mild VA dolichoectasia lesions have a smooth lumen and may have a benign natural history.^[35]^ Patients with moderate and severe VA dolichoectasia with stenosis, fusiform dilation, or an aneurysmal protrusion may have a worse natural history.^[36,37]^

### 5.3. Aneurysm associated with VA fenestrations

Aneurysms within VA fenestrations are saccular,^[38]^ whereas those on the fenestrated limb are dissected.^[39]^ Owing to their rarity, no studies have specifically investigated the natural history of these lesions. That of saccular lesions may be similar to the natural history of aneurysms at the VA–PICA junction, while dissecting lesions may have a similar natural history to that of dissecting aneurysms on the VA trunk.

## 6. EVT indications and options

### 6.1. EVT indications

EVT may be indicated for intracranial VA aneurysms with a poor natural history. The PHASES score (P: population, H: hypertension, A: age, S: size of aneurysm, E: early subarachnoid hemorrhage from another aneurysm, S: site of aneurysm) may be helpful for determining treatment.^[40]^ A high PHASES score may indicate the need for EVT. Alternatively, close follow-up is reasonable for such lesions.^[34,41]^

### 6.2. EVT options

**Figure 4.**
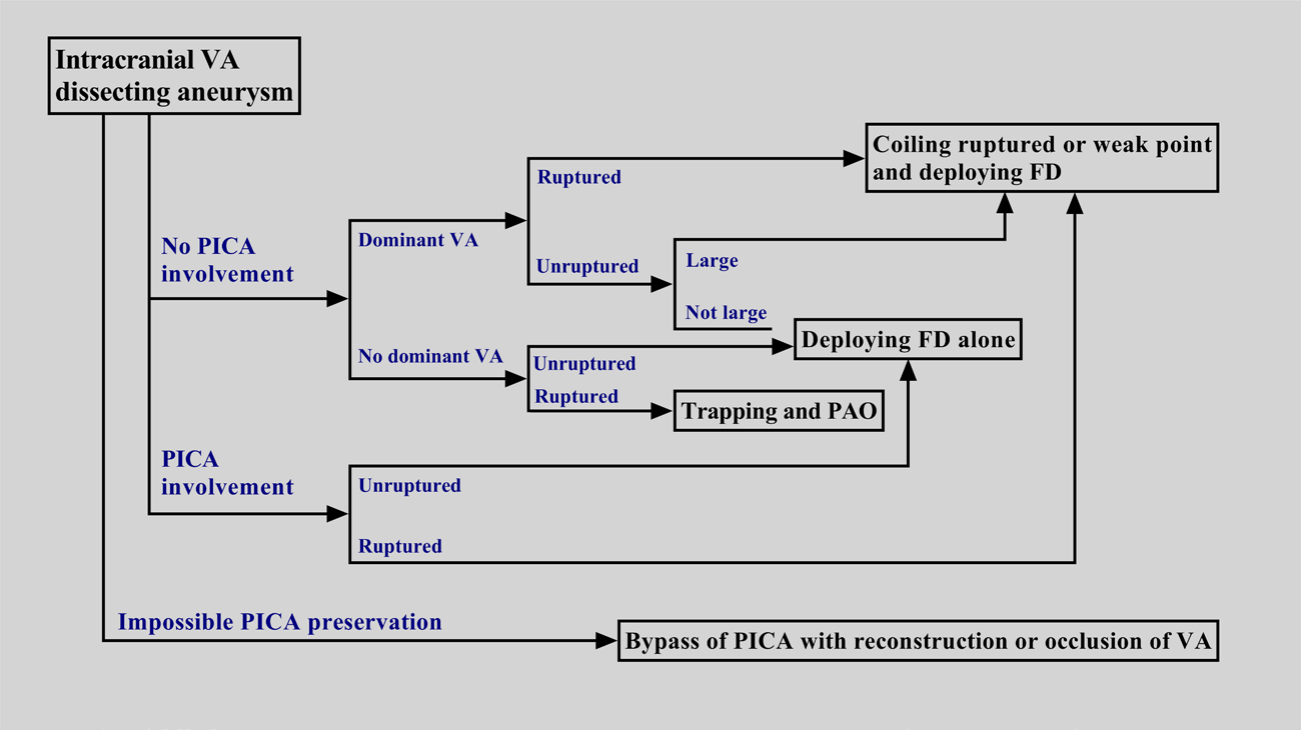
Flowchart for selecting the method of endovascular treatment for intracranial VA dissecting aneurysms. FD = flow diverter, PAO = parent artery occlusion, PICA = posterior inferior cerebellar artery, VA = vertebral artery.

EVT approaches for managing intracranial VA aneurysms include reconstructive or deconstructive approaches (Fig. 4). Reconstructive EVT is typically the first choice, as its goal involves preservation of the VA and PICA. For saccular aneurysms at the VA–PICA junction and within a fenestration, reconstructive EVT is often feasible. However, curing intracranial VA dissecting aneurysms with reconstructive EVT alongside traditional stent placement may be difficult. Placement of an FD has shown favorable safety and efficacy in treating these lesions and even those that have ruptured.^[42,43]^

Although FD deployment minimizes the use of deconstructive EVT, this latter treatment can still be administered for lesions at the nondominant VA and with no PICA involvement, especially for ruptured aneurysms. If the VA with the aneurysm is sacrificed, the contralateral VA can still provide sufficient blood supply to the vertebrobasilar arteries. It is essential to consider the collateral circulation from the anterior circulation, however. If the posterior communicating artery is fetal and the P1 segment of the posterior cerebral artery is hyperplastic, the vertebrobasilar arteries can achieve greater blood flow.^[44]^

## 7. Endovascular treatment techniques for different aneurysms

### 7.1. *Saccular aneurysm at the VA–PICA junction*

**Figure 5.**
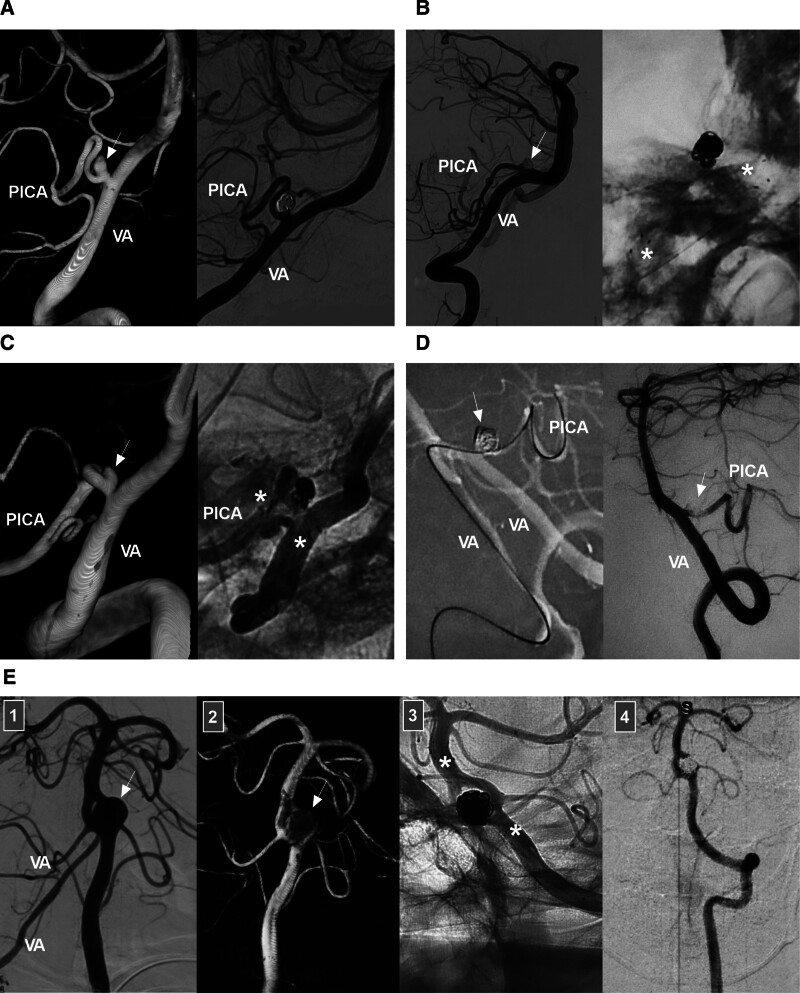
Endovascular treatment of aneurysms at the VA-PICA junction. (A) Coiling alone: left panel: DSA image showing an aneurysm (arrow) at the VA-PICA junction; right panel: follow-up DSA image showing complete occlusion of the aneurysm. (B) Stent-assisted coiling: left panel: DSA image showing a coiled saccular aneurysm (arrow) at the VA-PICA junction; right panel: X-ray image showing a stent (asterisks) deployed in the VA to cover the origin of the PICA. (C) Coiling assisted by antegrade stenting from the VA to the PICA: DSA image showing an aneurysm (arrow) at the VA-PICA junction; right panel: Unsubtracted DSA image showing coiling of the aneurysm via stenting (asterisks) from the ipsilateral VA to the PICA. (D) Coiling assisted by retrograde stenting from the contralateral VA to the PICA: Roadmap image showing a recurrently coiled saccular aneurysm (arrow) at the VA-PICA junction; the PICA was catheterized from the contralateral VA to await delivery of the stent; right panel: DSA image showing the coiled aneurysm (arrow). (E) panels 1 and 2: DSA and its reconstructive images showing a saccular aneurysm (arrows) in a VA fenestration; panel 3: DSA image showing coiling of the aneurysm with stenting assistance (asterisks); panel 4: follow-up DSA image showing complete occlusion of the aneurysm. DSA = digital subtraction angiography, PICA = posterior inferior cerebellar artery, VA = vertebral artery.

For aneurysms with a narrow neck, selective coiling is preferred (Fig. 5A). For aneurysms with a wide neck, selective coiling may be an option but may require temporary balloon assistance in the VA or permanent stent assistance. The balloon can be replaced by a Comaneci device (Rapid Medical, Yokneam, Israel), a new temporary bridging device that covers the aneurysm neck during coiling; after coiling, the device can be retrieved.^[45]^ If stent assistance is used, in most cases, only stenting in the VA is sufficient to spare the PICA (Fig. 5B). If stenting in the VA is unable to spare the PICA, antegrade stenting from the ipsilateral VA to the PICA (Fig. 5C) or retrograde stenting from the contralateral VA to the PICA (Fig. 5D) should be performed.^[46]^ In highly selective cases, an FD can be used for aneurysms at the VA–PICA junction; by covering the PICA, the FD can induce intra-aneurysmal thrombosis and spare the PICA owing to the resulting pressure gradient.^[47,48]^

### 7.2. *Aneurysm* associated with *VA fenestrations*

For aneurysms associated with VA fenestrations, sacrifice of the fenestrated limb may be considered a treatment option; however, it is preferable to preserve both limbs, as they might give off brainstem perforators.^[39]^ Antegrade single stenting and Y-stenting-assisted coiling can be used (Fig. 5E). Rarely, retrograde horizontal single-stent delivery across the aneurysmal neck from the contralateral VA and VBJ may be considered to assist in coiling.^[49]^ If the aneurysm is large or giant, an FD can be deployed.

### 7.3. Dissecting aneurysms of the intracranial VA trunk without PICA involvement

#### 7.3.1. Deconstructive EVT

**Figure 6.**
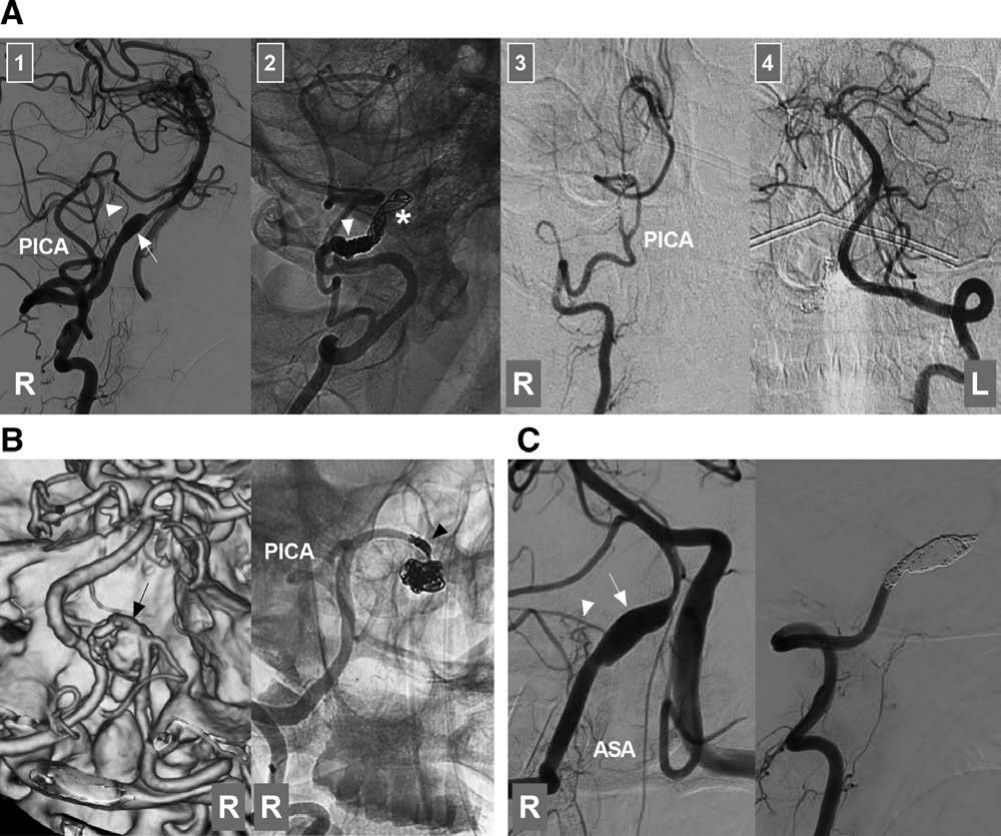
Deconstructive treatment for intracranial VA dissecting aneurysms without PICA involvement. (A) Proximal VA PAO: panel 1: DSA image showing a right VA dissecting aneurysm (arrow) and a perforating artery (arrowhead) emerging from the aneurysm; panel 2: Unsubtracted DSA image showing that the aneurysm (asterisk) is packed loosely and the proximal VA (arrowhead) is densely coiled; panels 3 and 4: 6-month follow-up DSA images of the bilateral VAs showing that the aneurysm has been cured, the right VA is supplying only the PICA, and the left VA is supplying the BA. The patient did not experience new neurologic deficits during and after treatment. (B) Trapping plus PAO: left panel: Computed tomography angiography showing a right VA dissecting aneurysm (arrow); right panel: DSA image showing trapping of the aneurysm and occlusion of the proximal VA (arrowhead). (C) Trapping with ischemia symptoms: left panel: DSA image showing a right VA dissecting aneurysm (arrow), a perforating artery (arrowhead) emerging from the aneurysm, and the ASA emerging from the end of the aneurysm; right panel, DSA image showing coiling of the aneurysm and occlusion of the perforating artery and ASA; postoperatively, the patient experienced symptoms of brainstem ischemia and recovered gradually but incompletely. ASA = anterior spinal artery, DSA = digital subtraction angiography, L = left, PAO = parent artery occlusion, PICA = posterior inferior cerebellar artery, R = right, VA = vertebral artery.

If the VA contralateral to the aneurysm gives off sufficient collaterals to the BA, deconstructive EVT can be used, including parent artery occlusion (PAO) proximal to the aneurysm (Fig. 6A) and aneurysm trapping plus PAO proximal to the aneurysm (Fig. 6B).^[1,50]^ Aneurysm trapping alone can result in anterograde recanalization of the aneurysm because of the water-hammer effect.^[51]^ Trapping and PAO is associated with a low aneurysmal recurrence rate, but ischemic complications may occur (Fig. 6C).^[6]^

When a VA aneurysm is at the pre-PICA position, after trapping the aneurysm, the PICA is supplied by the contralateral VA; however, this might cause brain ischemia due to hypoperfusion of the vessel. When a VA aneurysm is located in a post-PICA location, trapping the aneurysm can cause occlusion of the perforators because this segment often contains a large number of perforators, some of which are invisible on angiography.^[11,12,52]^ When a VA aneurysm is at the pre-PICA location, retrograde PAO from the contralateral VA can be used.^[53]^

When performing deconstructive EVT, it should be noted that intracranial VA occlusion may increase blood flow and hemodynamic stress in the contralateral VA, leading to the formation of new aneurysms.^[54]^ When performing deconstructive EVT for VA aneurysms, liquid embolic agents, such as the viscous Onyx HD-500 (Medtronic, Irvine, California, USA), can assist in coiling to finish the PAO.^[55]^

#### 7.3.2. Reconstructive EVT

##### 7.3.2.1. *Traditional stent-assisted EVT*

**Figure 7.**
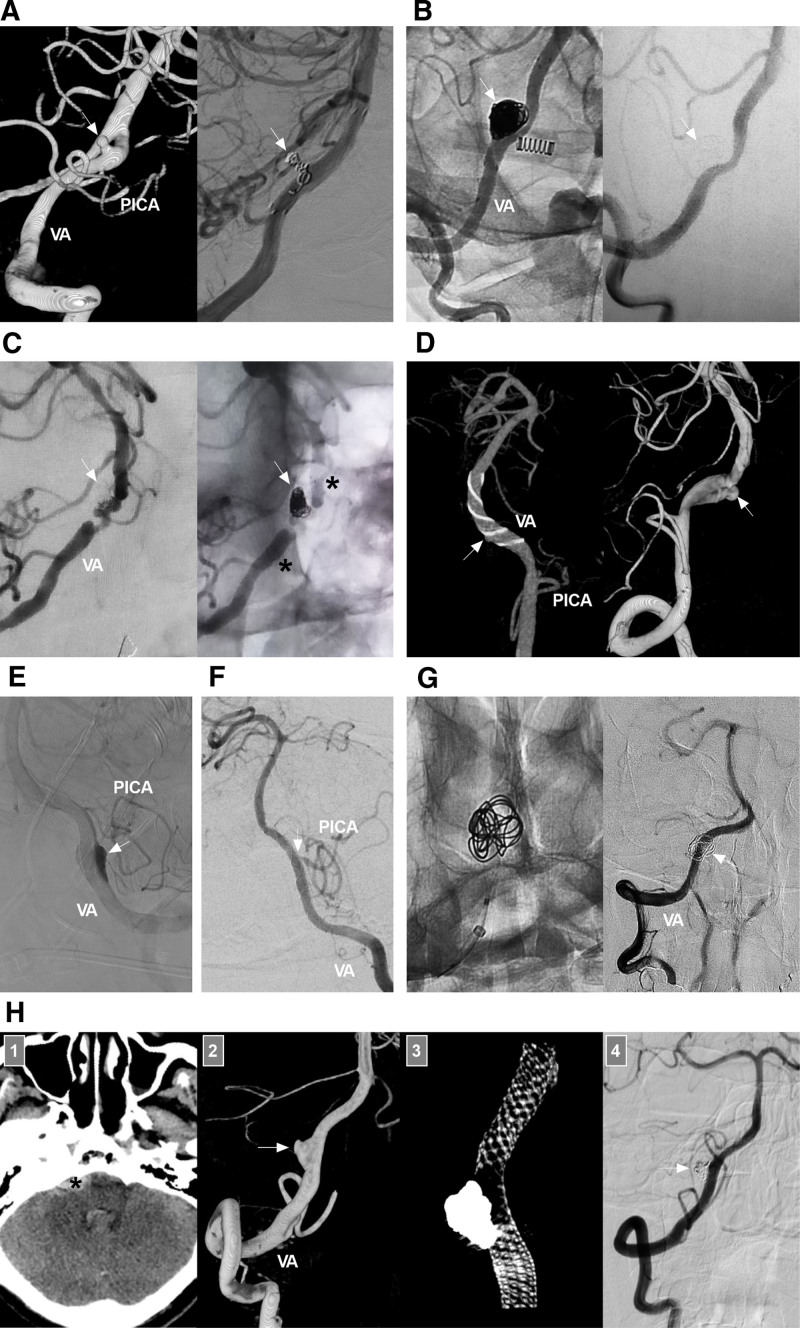
Reconstructive treatment for intracranial VA dissecting aneurysms without PICA involvement. (A) Traditional stent-assisted coiling: left panel: DSA image showing a small dissecting aneurysm of the VA post-PICA (arrow); right panel: DSA image showing coiling of the aneurysm (arrow) with stenting assistance. (B) Traditional stent-assisted coiling: left panel: unsubtracted DSA image showing a large VA dissecting aneurysm (arrow) that has been coiled with stenting assistance; right panel: follow-up DSA image showing cure of the aneurysm (arrow). (C) Neuroform Atlas stent-assisted coiling: left panel: DSA image showing a VA dissecting aneurysm (arrow) that has been coiled with stenting assistance; the parent VA has multiple tandem stenoses; right panel: unsubtracted DSA image showing the coiling (arrow) and stent markers (asterisks). (D) Recurrent VA dissecting aneurysm: left panel: reconstructive DSA image showing a VA dissecting aneurysm (arrow) covered by a LEO stent; right panel: follow-up DSA image showing aneurysm (arrow) recurrence. (E) FD deployment alone: DSA image showing contrast agent retention (arrow) in the aneurysm after FD deployment. (F) DSA image showing a narrowed PICA at the origin (arrow) after FD deployment. (G) FD deployment with loose coiling: left panel: X-ray image showing a VA dissecting aneurysm treated by FD deployment with loose coiling; right panel, follow-up DSA image showing cure of the aneurysm (arrow). (H) Ruptured aneurysm treated with FD deployment and coiling: panel 1: computed tomography image showing subarachnoid hemorrhage (asterisk); Panel 2: DSA image showing a VA aneurysm (arrow); Panel 3: vaso-reconstructive image showing the FD and coil; panel 4: follow-up DSA image showing that the aneurysm (arrow) has been cured. DSA = digital subtraction angiography, FD = flow diverter, PICA = posterior inferior cerebellar artery, VA = vertebral artery.

Most intracranial VA dissecting aneurysms do not have a narrow aneurysm neck, and permanent stent-assisted coiling is often necessary.^[5]^ Currently, traditional stents with low metal coverage, including the Enterprise stent (Codman Neurovascular, Raynham), Solitaire stent (Medtronic, Irvine), Neuroform EZ and Atlas stents (Stryker, Neurovascular, Fremont), LVIS (MicroVention Inc., Aliso Viejo), and LEO stent (Balt Extrusion, Montmorency, France), are widely used.^[56]^ For small and sidewall intracranial VA dissecting aneurysms, the protection provided by traditional stents is often sufficient (Fig. 7A and B).^[57]^ The low-profile Neuroform Atlas stent can be deployed more easily, which makes EVT a convenient method for managing aneurysms associated with stenosis (Fig. 7C). For ruptured, large or fusiform VA aneurysms, recurrence or incomplete occlusion may occur after traditional stent deployment (Fig. 7D).^[58]^ Overlapping deployment of multiple traditional stents may be a preferred modality for the treatment of VA dissecting aneurysms.^[59]^

When traditional stents with low metal coverage are deployed to assist in coiling intracranial VA aneurysms, antiplatelet management is necessary. For unruptured lesions, dual-antiplatelet medications of 100 mg aspirin and 75 mg clopidogrel for a minimum of 3 to 5 days before EVT can be administered. For ruptured lesions, a loading dose of 300 mg aspirin and 300 mg clopidogrel can be given for a minimum of 3 hours before EVT. After EVT, dual-antiplatelet medication can be continued for 3 to 6 months. Then, 100 mg of aspirin can be given for a minimum of 6 months or for life.^[60]^ When a Neuroform Atlas stent is used, the duration of dual-antiplatelet medications can be reduced. When an LEO stent is planned, clopidogrel can be replaced with ticagrelor to reduce the incidence of ischemic complications.

##### 7.3.2.2. *FD deployment*

Reconstructive EVT with FD deployment has shown favorable outcomes for patients with large, fusiform intracranial VA dissecting aneurysms or dolichoectatic aneurysms.^[61]^ During FD deployment, adjunctive coiling is not necessary for small or medium-sized lesions (Fig. 7E and F).^[62]^ However, for large, sidewall or ruptured lesions, adjunctive coiling may be necessary (Fig. 7G and H). For small ruptured VA dissecting aneurysms, FD deployment may be safe and effective^[63]^; however, more evidence is needed.

After the FD covers the PICA ostia, the fate of the PICA must be considered. In general, the PICAs could be occluded or narrowed (Fig. 7F).^[64]^ Liu et al’s meta-analysis revealed that the rate of occlusion/narrowing when the FD covered the PICA was 6.8%, and the risk of branch occlusion-related complications was low (incidence < 5%).^[65]^ Therefore, symptomatic PICA occlusion is uncommon after FD coverage.

When an FD is deployed to reconstruct the intracranial VA, antiplatelet management is mandatory. To date, there are no uniform criteria for antiplatelet/anticoagulation management. However, dual-antiplatelet therapy is necessary, but the medication time, drug type and dose vary. Mazur et al’s study investigating a pipeline embolization device deployment for unruptured VA aneurysms can serve as a reference for antiplatelet management; before EVT, patients were premedicated with dual-antiplatelet agents (aspirin 325 mg/day and clopidogrel 75 mg/day) for 7 days. During EVT, patients were given anticoagulant treatment in the form of an intravenous heparin bolus (70 U/kg) with titration to maintain an activated clotting time > 250 s or 2 times the patient’s baseline. After EVT, patients were premedicated with dual-antiplatelet agents for 6 months; in the follow-up angiography, if the aneurysm was occluded and the FD was patent, clopidogrel was discontinued, and aspirin was continued for life.^[66]^ For ruptured VA aneurysms scheduled for treatment with FD deployment, a loading dose of dual-antiplatelet therapy should be started before the procedure.^[67]^ However, the risk of perioperative hemorrhagic complications from antiplatelet management must be considered.

For intracranial dolichoectatic VA aneurysms, FD deployment with dual-antiplatelet therapy is often feasible but carries a high risk of perforator occlusion and brainstem compression. Therefore, triple medication therapy, including dual-antiplatelet therapy plus oral anticoagulation, may be needed to preserve the patency of brainstem perforators. Antithrombotic therapy can not only diminish the risk of ischemic stroke following perforating artery occlusion but also reduce aneurysm progression by decreasing the risk of thrombus formation between the FD and the aneurysm wall. Siddiqui et al reported that patients with dolichoectatic vertebrobasilar fusiform aneurysms treated with FD deployment in the triple therapy group had fewer ischemic strokes, less symptom progression, and overall better outcomes at the last follow-up than patients in the dual-antiplatelet therapy group did.^[68]^

In a Chinese multicenter cohort study of intracranial unruptured VA dissecting aneurysms treated with pipeline embolization devices in 2023, patients took dual-antiplatelet medication, including aspirin (100–300 mg daily) and clopidogrel (75 mg daily), 3 days before EVT and 6 months after EVT. Patients identified as clopidogrel nonresponders according to platelet function testing were given aspirin (100 mg/d) and ticagrelor (90 mg/twice daily).^[42]^ Single antiplatelet medication was maintained for 6 months to 1 year or life, depending on the patient’s clinical and radiological circumstances.^[69]^

On the basis of the experience in our institute, we can offer suggestions for antiplatelet/anticoagulation management for intracranial VA aneurysms. For patients with unruptured aneurysms, dual-antiplatelet agents (aspirin 100 mg/d and clopidogrel 75 mg/d) were given for 5 to 7 days before EVT. Patients identified as clopidogrel nonresponders were given aspirin (100 mg/d) and ticagrelor (90 mg/twice daily). During FD deployment, 3000 to 5000 U of heparin was administered intravenously as a bolus infusion. For patients with ruptured aneurysms, a minimum of 3 hours before EVT, a loading dose of dual-antiplatelet agents (aspirin 300 mg and ticagrelor 180 mg) was given. During FD deployment and aneurysm coiling, tirofiban (4–6 mL by intravenous bolus) was given. After EVT, tirofiban was continued for 24 hours via continuous intravenous pumping (4–6 mL/h). From the second day after EVT, for all patients, dual-antiplatelet agents (aspirin and clopidogrel or aspirin and ticagrelor) were continued for 6 months, and then clopidogrel or ticagrelor was discontinued, but treatment with aspirin was continued for 6 months to 1 year or for life on the basis of the follow-up angiographic outcomes.

##### 7.3.2.3. Cover stent deployment

For intracranial VA dissecting aneurysms without PICA involvement, reconstructive EVT using a covered stent is an attractive alternative for definitive treatment. However, the covered stent may occlude the perforating arteries of the VA. Sometimes, the apposition of the covered stent is insufficient for excluding blood flow into the aneurysm; therefore, covered stents should be used in highly selective cases. In addition, the covered stent is often too stiff, and therefore delivering and passing it through a tortuous VA loop can be difficult.^[70]^

### 7.4. Dissecting aneurysms of the intracranial VA trunk with PICA involvement

The PICA is involved in 14.6% of intracranial VA dissecting aneurysms.^[4,71]^ PICA involvement was found to be an independent risk factor for intracranial VA aneurysm recurrence and incomplete occlusion.^[50]^

#### 7.4.1. Deconstructive EVT

PAO of the VA under the aneurysm can be used to treat intracranial VA trunk aneurysms with PICA involvement. After proximal PAO, the PICA is preserved, and the hemodynamic stress in the aneurysm decreases. However, aneurysms can still obtain blood from the contralateral VA, leading to progression or rupture. In addition, the PICA territory can experience ischemia due to hypoperfusion from insufficient retrograde blood flow. The most reliable treatment is achieved with aneurysm trapping plus proximal PAO, but patients who can tolerate PICA obliteration must be carefully selected, as sacrificing the PICA can lead to brainstem and cerebellum ischemia. Therefore, aneurysm and VA trapping plus preservation of the PICA is an option; in select patients, trapping the aneurysm with ipsilateral or contralateral VA-PICA stenting can preserve the PICA.^[72]^

#### 7.4.2. Reconstructive EVT

**Figure 8.**
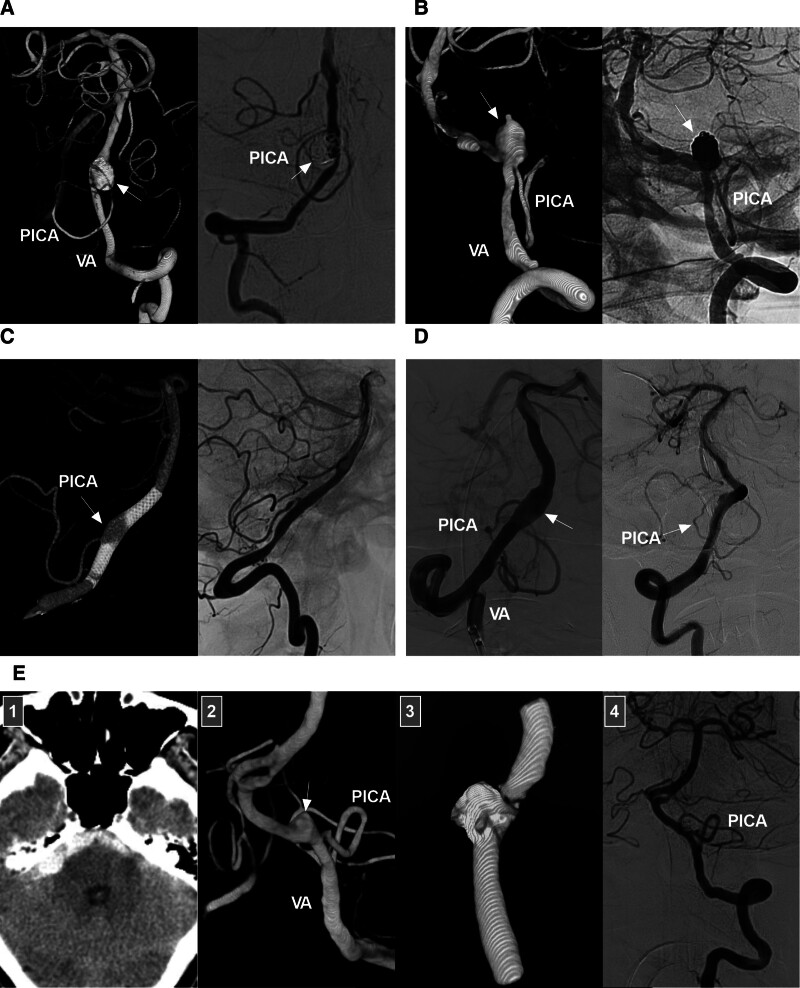
Reconstructive treatment for VA dissecting aneurysms with PICA involvement. (A) Traditional stent-assisted coiling: follow-up 3-dimensional (left panel) and 2-dimensional (right panel) DSA images showing complete occlusion of a sidewall VA trunk dissecting aneurysm (arrow) with PICA involvement; the PICA has been preserved. (B) Traditional stent-assisted coiling: left panel: 3-dimensional DSA image showing a saccular VA trunk aneurysm (arrow) with PICA involvement; right panel: unsubtracted DSA image showing that the aneurysm (arrow) has been coiled with stenting assistance; the PICA has been preserved. (C) FD deployment alone: left panel: reconstructive DSA image showing coverage of the aneurysm (arrow) by an FD; the PICA is patent; right panel: 1-year follow-up DSA image showing cure of the dissecting aneurysm and preservation of the PICA. (D) FD deployment alone: left panel: DSA image showing a VA dissecting aneurysm (arrow) following FD coverage; the PICA (arrow) is patent; right panel: 6-month follow-up DSA image showing reconstruction of the VA, regression of the aneurysm (arrow), and patency and thinning of the PICA. (E) FD deployment with adjunctive coiling: panel 1: Computed tomography image showing subarachnoid hemorrhage (asterisk); panel 2: 3-dimensional DSA image showing a VA dissecting aneurysm (arrow), with the PICA (arrowhead) emerging from the proximal portion of the aneurysm; Panel 3: Image showing the FD device and coils; Panel 4: 6-month follow-up DSA image showing cure of the aneurysm and patency of the PICA. DSA = digital subtraction angiography, FD = deployment, PICA = posterior inferior cerebellar artery, VA = vertebral artery.

Reconstructive EVT for occluding the aneurysm and preserving the VA and PICA is an ideal option.^[73]^ This technique usually requires permanent stenting assistance, including with traditional stents and FDs. During reconstructive EVT with traditional stents, to preserve the PICA, dense coiling of the aneurysm is necessary (Fig. 8A and B). However, for most VA aneurysms with PICA involvement, the aneurysmal sac must be left partially or entirely open to ensure adequate PICA flow, which leads to a high risk of progression or rebleeding. Under these circumstances, traditional stents are not appropriate because they result in insufficient flow redirection away from the aneurysm; therefore, FDs must be used.

FDs offer a favorable balance between securing aneurysms and preventing infarction due to branch and perforator impairment. After FD deployment, thrombi can rapidly form in the aneurysm, and endothelialization subsequently occurs; finally, the endothelium shrinks and collapses around the FD while the PICA and perforating branches are preserved.^[5]^ In unruptured VA trunk dissecting aneurysms with PICA involvement, FDs can be used alone (Fig. 8C and D). However, in ruptured lesions, targeted coiling of the ruptured bleb is necessary, after which the FD can be deployed (Fig. 8E).^[74]^

### 7.5. Bilateral intracranial VA dissecting aneurysms

**Figure 9.**
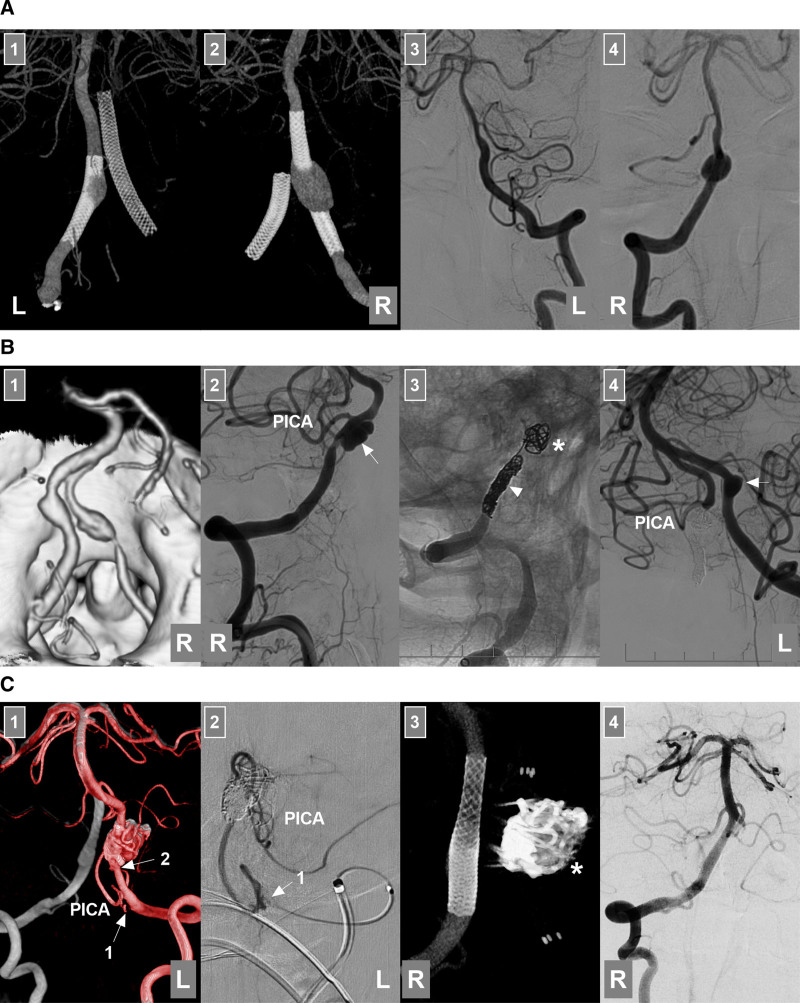
EVT of bilateral intracranial VA dissecting aneurysms. (A) FD deployment for bilateral VA dissecting aneurysms: panels 1 and 2: vaso-reconstructive DSA images showing 2 VA aneurysms treated with FD deployment; panel 3 and 4: 1-year follow-up DSA image showing cure of the left but not the right aneurysm. (B) PAO for ipsilateral ruptured VA dissecting aneurysms: panel 1: computed tomography angiography image showing bilateral VA dissecting aneurysms; panel 2: DSA image showing a right VA dissecting aneurysm (arrow) at the PICA origin; panel 3: Unsubtracted DSA image showing loose coiling of the right aneurysm (asterisk) and complete occlusion of the proximal VA (arrowhead); panel 4: DSA image showing a right PICA supplied retrogradely by the left VA; plans were made to treat the left aneurysm (arrow) later. (C) Simultaneous deconstructive and reconstructive EVT: panel 1: reconstructive 3-dimensional DSA image showing bilateral VA dissecting aneurysms, the left of which recurred after coiling assisted by traditional stenting, while the left PICA has a double origin (Arrows with numbers 1 and 2); panel 2: microcatheter angiography in the lower origin of the PICA (arrow with number 1) showing the course of the vessel; panel 3: vaso-reconstructive DSA image showing coil occlusion of the left aneurysm (asterisk) and FD deployment to cover the right aneurysm; panel 4: 6-month follow-up DSA image of the right VA showing cure of the right aneurysm and sufficient blood flow to the vertebrobasilar arteries from the right VA. DSA = digital subtraction angiography, EVT = endovascular treatment, FD = flow diverter, L = left, PAO = parent artery occlusion, PICA = posterior inferior cerebellar artery, R = right, VA = vertebral artery.

Bilateral intracranial VA dissecting aneurysms are rare^[75]^; nevertheless, several EVT options exist for these lesions. For reconstructive EVT, bilateral FD deployment, either staged or simultaneous, might enable a greater rate of complete aneurysm occlusion (Fig. 9A).^[22]^ Bilateral VA occlusion can also be considered; however, it not tolerated by most patients. Additionally, occlusion of only 1 VA may cause growth of the contralateral aneurysm due to increased flow. PAO in the hypoplastic VA and reconstructive EVT via either staged or simultaneous FD deployment in the dominant VA can be chosen (Fig. 9B and C).^[76]^ If bilateral FD deployment or unilateral FD deployment plus ipsilateral PAO is performed, the dual-antiplatelet agent regimen must be chosen carefully to avoid ischemic complications.

### 7.6. Recurrent intracranial VA dissecting aneurysms

Intracranial VA dissecting aneurysms can recur after EVT due to low metal coverage, traditional stent deployment or incomplete aneurysm trapping, especially in large aneurysms.^[77]^ The recurrence rate for these lesions after routine EVT ranges from 13% to 33%.^[78]^ For recurrent aneurysms, PAO can be considered an option in patients who can tolerate sacrifice of the parent VA (Fig. 9C). FD deployment can also be an effective option for lesions with prior stent-assisted coiling EVT.^[79]^ However, it may be difficult for microcatheters to pass the prior stent lumen due to poor stent visualization and deformation, especially in patients with long fusiform or large circumferential dissecting aneurysms.^[5]^

## 8. Outcomes and complications

For patients with intracranial VA aneurysms, EVT can result in a good neurologic outcomes (modified Rankin scale (mRS) score of 0–2) and adequate aneurysm occlusion (complete occlusion and nearly complete occlusion (>90% occlusion with a small neck remnant)).^[58]^

### 8.1. *Saccular aneurysms at the VA–PICA junction*

To date, no exact data concerning the outcomes of patients who have undergone EVT for saccular aneurysms at the VA–PICA junction have been reported. The data may be similar to the result of Ali et al’s meta-analysis.^[80]^ In Ali et al’s meta-analysis including 455 saccular PICA aneurysms, EVT was performed for 300 aneurysms, the rate of adequate aneurysm occlusion was 69.1%, and 77.6% of patients achieved good neurologic outcomes. However, Ali et al’s meta-analysis included some aneurysms of the PICA trunk; therefore, the true rates of adequate aneurysm occlusion and good neurologic outcomes after EVT for saccular aneurysms at the VA–PICA junction may have been underestimated, as because PICA trunk aneurysms are dissecting aneurysms, which have worse clinical and angiographic prognoses than saccular aneurysms at the VA–PICA junction.

**Figure 10.**
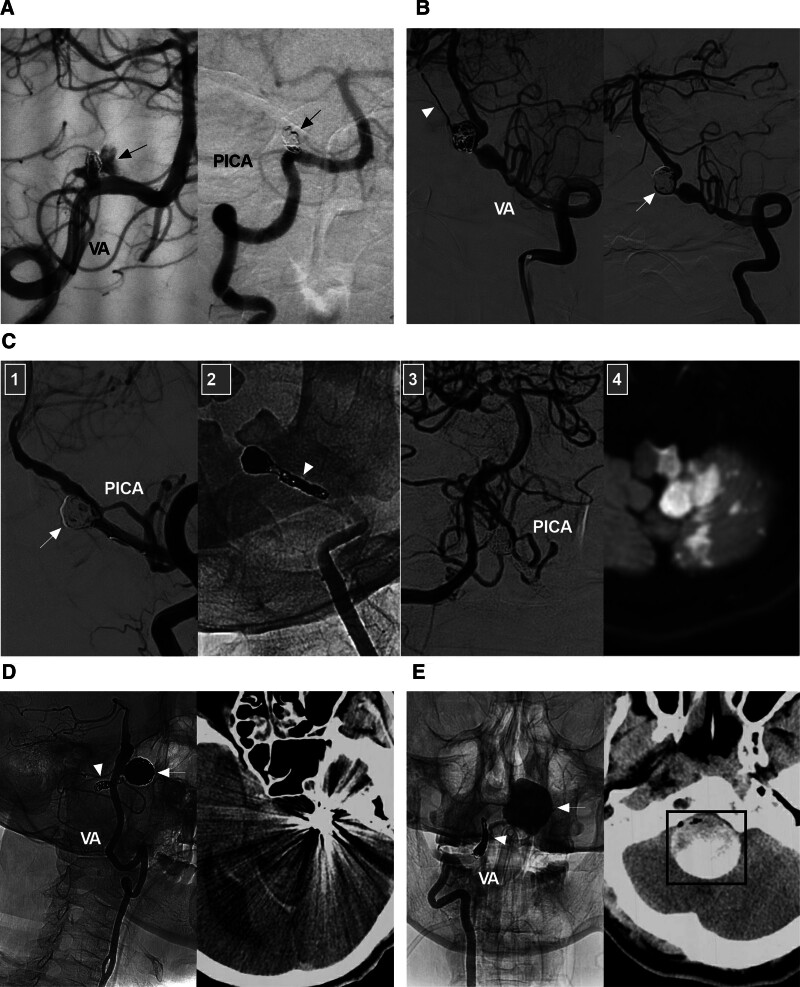
Complications of traditional coiling EVT. (A) Hemorrhagic complications: left panel, DSA image showing an aneurysm at the VA and PICA junction that ruptured during coiling and contrast agent extravasation (arrow); right panel: follow-up DSA image showing complete aneurysm occlusion (arrow). (B) Hemorrhagic complications: left panel: DSA image showing a VA dissecting aneurysm perforated by the microcatheter and a coil loop (arrowhead). right panel: DSA image showing complete coiling of the aneurysm (arrow). (C) Ischemic complications: panel 1: DSA image showing coiling of an aneurysm (arrow) with PICA involvement; panel 2: Unsubtracted DSA image showing occlusion of the VA (arrowhead) proximal to an aneurysm; panel 3: DSA of a contralateral VA showing irrigation of the PICA on the side of the aneurysm by retrograde blood flow; panel 4: postoperative magnetic resonance diffuse sequence image showing acute infarction of the PICA territory. (D) Mild brainstem compression due to a mass effect: left panel: unsubtracted DSA image showing a large VA aneurysm treated by aneurysm trapping (arrow) and proximal PAO (arrowhead); right panel: postoperatively, a patient who experienced hemiplegia shows no hemorrhage on CT imaging. 1 week later, the patient had recovered. (E) Severe brainstem compression due to a mass effect: unsubtracted DSA image showing a large VA aneurysm (arrow) treated by proximal PAO (arrowhead); right panel: 1 day after EVT, the patient experienced respiratory arrest; CT image shows a highly dense aneurysm with a mass effect due to thrombosis (frame); 3 days later, the patient died. CT = computed tomography, DSA = digital subtraction angiography, EVT = endovascular treatment, PAO = parent artery occlusion, PICA = posterior inferior cerebellar artery, VA = vertebral artery.

In general, EVT for saccular aneurysms at the VA–PICA junction yields a > 80% rate of adequate aneurysm occlusion and a >80% rate of good neurologic outcomes.^[81–83]^ However, EVT for aneurysms at the VA–PICA junction is associated with unavoidable procedure-related hemorrhagic and ischemic complications. The rates of these complications vary across different reports, ranging from 10% in Mericle et al’s study^[23]^ to 19% in Garner et al’s study^[83]^ and 21% in Peluso et al’s study.^[84]^ A small aneurysm size is a risk factor for intraoperative aneurysmal rupture due to catheterization difficulty. In addition, when the slack microcatheter is pushed through the VA loop, the microcatheter may jump upward, perforating the aneurysm (Fig. 10A).^[84]^ Procedure-related ischemic complications may result from injury to or occlusion of branch vessels or perforators.^[83,84]^

For aneurysms at the VA–PICA junction, the use of an FD to cover the origin of the PICA can yield good results.^[48]^ Liu et al performed a meta-analysis of 46 PICA aneurysms treated via FD deployment, primarily aneurysms at the VA–PICA junction. During a follow-up period of 6.5 to 19.5 months, the technical success rate was 100%, the complete aneurysm occlusion rate was 81%, the FD-related complication rate was 18%, 1 patient died of rebleeding, and the mortality rate was < 1%.^[47]^

### 8.2. Aneurysm associated with intracranial VA fenestrations

With selective or stent-assisted coiling, aneurysms associated with VA fenestrations can achieve good outcomes.^[49,85]^

### 8.3. Intracranial VA dissecting aneurysms

#### 8.3.1. Patient outcomes

Deconstructive and reconstructive EVT, including PAO, stent-assisted coiling and FD deployment, are effective for treating intracranial VA dissecting aneurysms.^[22,58,86–89]^ EVT can yield a >80% rate of adequate aneurysm occlusion and a > 85% rate of good clinical outcome.^[64,90,91]^ Lee et al reported no significant differences in clinical outcomes or ischemic complications between PAO and traditional stent-assisted treatment for ruptured intracranial VA dissecting aneurysms.^[86]^ Oh et al compared traditional stent-assisted coiling with FD deployment in patients with unruptured intracranial VA aneurysms and reported that both methods were safe and effective.^[6]^ However, traditional stent-assisted coiling may be associated with recurrence, with rates ranging from 5% to 10%, especially for fusiform lesions and those with PICA involvement.^[30,90]^ FD deployment can also reduce the likelihood of recurrence.^[42,64]^

Currently, FD deployment is the first choice for intracranial VA dissecting aneurysms. In 2024, Liu et al performed a systematic review and meta-analysis including 541 intracranial VA aneurysms treated with FDs. FD deployment was associated with a favorable clinical outcome rate of 95%, a complete aneurysmal occlusion rate of 81%, an ischemic complication incidence of 4%, a hemorrhagic complication incidence of 1%, a PICA preservation rate of 95%, and an in-stent stenosis or occlusion rate of 6% (95% CI, 3%–10%) across clinical and angiographic follow-up periods of 13.6 months and 11.9 months, respectively.^[64]^

When traditional stents are used, the placement of multiple stents may play a key role in achieving favorable long-term radiological outcomes.^[58,59]^ In Kim et al’s report, of 146 patients with VA dissecting aneurysms, 25 (17.1%) were managed with FDs, and 121 (82.9%) were managed with overlapping stents. Of the 124 patients with ≥ 12 months of angiographic follow-up (median 33 months), 22 (17.7%) patients were managed with FDs, 19 (15.3%) were managed with double stents, and 83 (66.9%) were managed with triple stents. The rate of favorable outcomes for patients with FDs was 81.8%, and that for patients with overlapping stents was 98.8%. Double stents and triple stents were not significantly associated with favorable outcomes with respect to FDs after adjusting for thrombosed aneurysms, aneurysm shape, nondominant VA, PICA involvement, and procedure type. FD deployment in patients with partially thrombosed aneurysms may be associated with favorable angiographic outcomes.^[59]^

#### 8.3.2. Complications

Endovascular treatment of intracranial VA dissecting aneurysms may be associated with hemorrhagic and ischemic complications and deterioration of the mass effect (Fig. 10B–E).^[92]^ According to Lee et al’s report, the rate of symptomatic ischemic complications, for which intra-aneurysmal thrombosis and PICA involvement may be independent risk factors, was 13.4%.^[93]^

In deconstructive EVT, a long trapping segment, VA occlusion distal to the PICA, and a dominant VA are associated with ischemic complications.^[94]^ According to previous reports, the incidence of medullary infarction due to occlusion of the perforating arteries during PAO ranges from 19% to 47%.^[95,96]^ Intracranial VAs without a PICA may give off a significantly larger number of perforators, indicating that trapping this type of intracranial VA can be associated with ischemic complications.^[95]^

**Figure 11.**
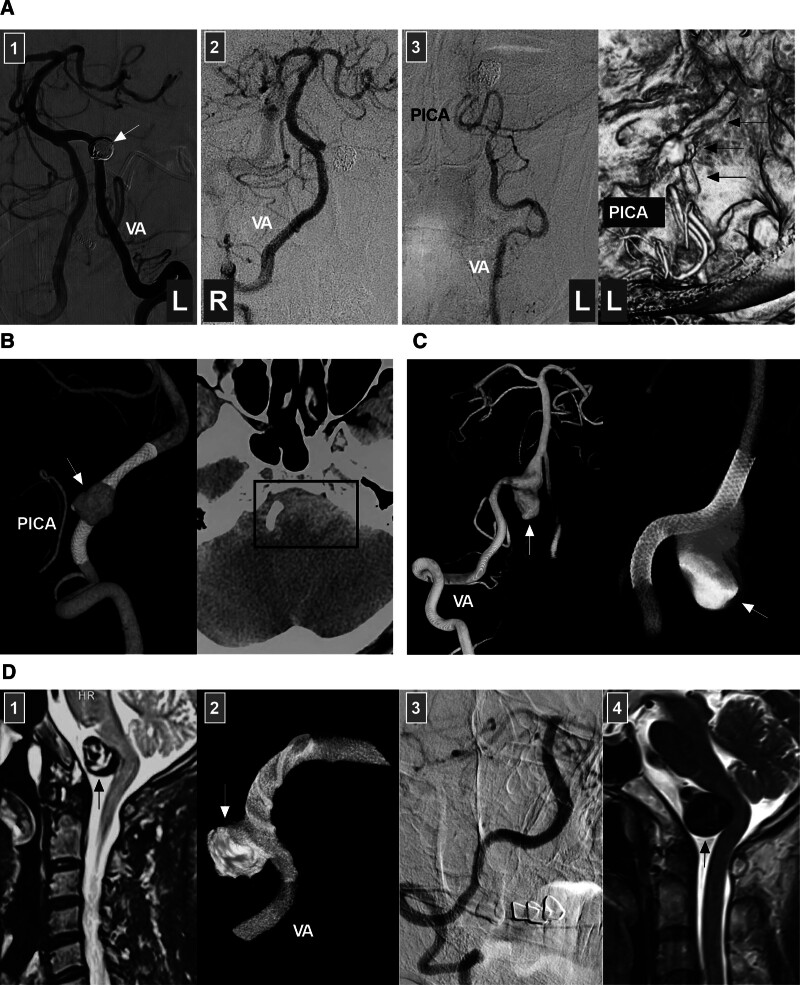
Complications of FD deployment. (A) VA occlusion after FD deployment: panel 1: DSA image of the left VA showing a coiled VA aneurysm (arrow) covered by an FD; panel 2: 6-month follow-up DSA image of the right VA showing that the vessel supplies the vertebrobasilar arteries; panel 3: DSA image of the left VA showing that the vessel terminates at the PICA and that the VA distal to the PICA is occluded (left panel); Reconstructive DSA image showing the FD device (arrows) and coils in the occluded left VA (right panel). (B) Hemorrhagic complications: left panel: vaso-reconstructive DSA image showing a VA dissecting aneurysm (arrow) treated with FD deployment; right panel: 6 h after EVT, computed tomography imaging shows subarachnoid hemorrhage (frame), indicating aneurysm rupture. (C) VA aneurysm with a mass effect after FD deployment: left panel: DSA image showing a large intracranial VA aneurysm (arrow); right panel: vaso-reconstructive DSA image showing FD coverage of the aneurysm (arrow). Postoperatively, the patient experienced weakness in the left upper and lower limbs, while MRI showed no hemorrhage or infarction. Three days later, the patient recovered, indicating that the aneurysm had expanded after FD deployment. (D) VA aneurysm with mass effect after FD deployment: panel 1: MRI showing a lesion (arrow) with a mass effect to the brainstem; panel 2: vaso-reconstructive DSA image showing the aneurysm (arrow) covered by the FD. panel 3: 6-month follow-up DSA image showing complete aneurysm occlusion; panel 4: follow-up MRI showing that the aneurysm (arrow) has not shrunk. DSA = digital subtraction angiography, MRI = magnetic resonance image, PICA = posterior inferior cerebellar artery, VA = vertebral artery.

FD deployment for intracranial VA dissecting aneurysms is also associated with complications.^[42,91]^ In Liu et al’s meta-analysis, FD deployment for the treatment of VA dissecting aneurysms was associated with a 4% rate of ischemic complications and a 1% rate of hemorrhagic complications.^[64]^ Ischemic complications included in-stent thrombosis or VA occlusion (Fig. 11A). Ramirez-Velandia et al reported that thromboembolic events presenting with large-vessel occlusion tended to occur in vessels of the vertebrobasilar system.^[97]^ Among hemorrhagic complications, delayed rupture is particularly disastrous (Fig. 11B). After FD deployment, the possibility of progressive mass effects must be considered (Fig. 11C and D).^[32]^

## 9. Development of new techniques

### 9.1. New radial access products

The BENCHMARK™ BMX®81 System (Penumbra, Inc., Alameda) is a recently developed guiding catheter whose design and size support both radial and femoral access across a wide range of neurovascular procedures. It includes a 7F large-bore access catheter with a 0.081” bore diameter.^[98]^ The ease of use and versatility of this new catheter allows it to serve as an alternative for achieving transfemoral access as well as to other catheters used for radial access.^[99]^ The catheter is helpful for administering EVT for intracranial VA aneurysms.

### 9.2. New and promising FDs

New generations of FDs with surface modifications have recently been introduced, including the Pipeline^TM^ Flex with Shield Technology, Pipeline^TM^ Vantage with Shield Technology, FRED^TM^ X, Phenox HPC and Acandis Dervio^®^ 2 devices.^[100,101]^ These new products can eventually facilitate single antiplatelet therapy.^[102,103]^ Drug-eluting FDs represent a promising avenue for reducing thrombogenicity.^[104]^ Bio and hemocompatible surface modifications for FDs as well as bioresorbable FDs are in development.^[105]^

### 9.3. Stent-like devices and intrasaccular flow disruptors

Stent-like devices currently in use for treating intracranial VA aneurysms include the pCONus device (Phenox GmbH, Bochum, Germany), PulseRider device (Cerenovus, Irvine), Barrel device (Medtronic/Covidien, Irvine), and eCLIP (Endovascular Clip System) device (Evasc Medical Systems, Vancouver, Canada).^[106]^ In addition to the Woven EndoBridge (WEB) device (Aliso Viejo), other intrasaccular flow disruptors can also be used to treat aneurysms at the VA–PICA junction or within the fenestration, such as the Medina Embolic Device (Medtronic, Irvine), Contour device (Cerus Neurovascular, Fremont) and Luna/Artisse embolization system (Medtronic, Irvine).^[107–111]^

### 9.4. Promising techniques

There are several promising techniques for administering EVT for intracranial VA aneurysms. Virtual reality simulation techniques with stent planning software can assist neuroradiologists in performing EVT.^[112]^ The use of artificial intelligence (AI) and robotic systems may improve the use of EVT for intracranial VA aneurysms.^[113]^ AI systems could be used to analyze angiographic images in real time to suggest optimal locations for stent placement, thereby minimizing procedural complications and improving clinical outcomes. Similarly, robotic catheter systems can precisely navigate intricate vascular anatomy, enabling clinicians to accurately deploy FDs for anatomically challenging VA aneurysms. In addition, transcriptomic studies and gene therapy can be helpful for managing intracranial VA aneurysms.^[105,114]^

## 10. The state of surgery in treating intracranial VA aneurysms

**Figure 12.**
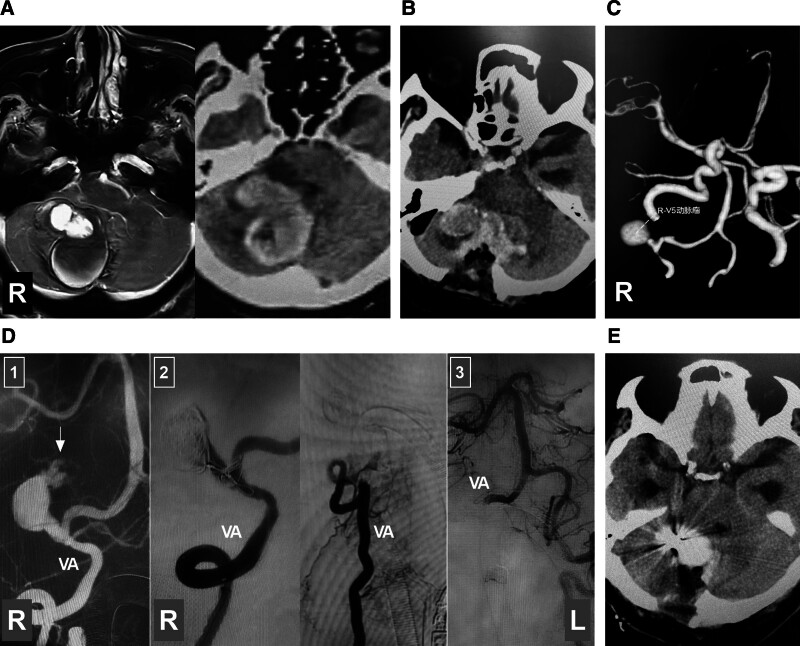
Combined EVT and aneurysmectomy in treating a giant intracranial VA aneurysm. (A) Enhanced magnetic resonance (left panel) and CT (right panel) images showing a lesion with a mass effect in the posterior fossa. (B) CT image showing the area of interest after exploratory craniotomy. (C) CT angiography image confirming that the lesion is an aneurysm. (D) Panel 1: Roadmap image showing the rupture point (arrow) of a giant VA aneurysm; panel 2: DSA image of the right VA showing occlusion of the aneurysm and parent VA with coils; panel 3: DSA image of the left VA showing that the vessel supplies the right VA distal to the aneurysm and basilar artery. (D) Postoperative CT showing the results of aneurysmectomy and resolution of the mass effect. CT = computed tomography, DSA = digital subtraction angiography, L = left, R = right, VA = vertebral artery.

With the development of EVT techniques and products, open surgery is undeniably becoming less common. Open surgery is challenging in the treatment of intracranial VA aneurysms because of their deep locations and complex anatomies and the involvement of important branches. In general, open surgery can be used only in select cases where extracranial-intracranial bypass and aneurysmectomy are necessary. Bypass arrangements include the high-flow radial artery graft-VA bypass or the low-flow occipital artery-PICA bypass.^[2]^ For giant thrombotic VA aneurysms with mass effects, aneurysmectomy with EVT assistance is necessary (Fig. 12).

## 11. Conclusion

Deconstructive and reconstructive EVT can be used to manage intracranial VA aneurysms. The key to successful EVT is preservation of the PICA and avoidance of injury to brainstem perforators. FD deployment plays an important role in repairing the intracranial VA. In general, after appropriate patients are selected, EVT can result in good outcomes. However, the potential for EVT-related complications should be taken into account. In addition, new products and techniques show promise for achieving successful EVT of intracranial VA aneurysms. In select cases, extracranial-intracranial bypass and aneurysmectomy are necessary.

## Author contributions

**Conceptualization:** Jinlu Yu.

**Data curation:** Yanming Qu, Jinlu Yu.

**Software:** Yanming Qu, Jinlu Yu.

**Supervision:** Jinlu Yu.

**Writing – original draft:** Yanming Qu, Jinlu Yu.

**Writing – review & editing:** Yanming Qu, Jinlu Yu.
